# Brain imaging studies in Internet Gaming Disorder (IGD) and problematic social network site use

**DOI:** 10.1192/j.eurpsy.2024.52

**Published:** 2024-08-27

**Authors:** A. Weinstein

**Affiliations:** Psychology, Ariel Univesrity, Ariel, Israel

## Abstract

**Abstract:**

Gaming disorder is characterized by ICD 11 as persistent or recurrent gaming behavior manifested by impaired control over gaming, increasing priority given to gaming over other life interests and daily activities; and continuation or escalation of gaming despite the occurrence of negative consequences. IGD shares to a large extent neurobiological alterations seen in other addictions, such as activation in brain regions associated with reward, reduced activity in impulse control areas and impaired decision-making; and reduced functional connectivity in brain networks that are involved in cognitive control, executive function, motivation and reward. Moreover, there were structural changes, mainly reduction in gray matter volume and white matter density. Comorbidity studies indicate that executive control networks in ADHD may increase the susceptibility to develop IGD. Problematic SNS use has been associated with an increased rate of depression, anxiety, stress, obsessive-compulsive disorder (OCD), attention-deficit/hyperactivity disorder (ADHD), and propensity to excessive alcohol use. It may also lead to vulnerability to aggression, cyberbullying and fear of missing out (FOMO). There is little evidence for cognitive impairments, but there is some preliminary event-related potentials (ERPs) evidence for inefficiency in allocating and monitoring resources and inhibitory control. There is evidence for reduced sleep quality and quantity, longer sleeping latency and more sleep disturbance. Brain imaging studies showed impaired inhibitory-control mechanism, reduced gray matter volumes in the nucleus accumbens, amygdala, and the insula, suggesting rewarding effects of SNS use on the brain.

**Disclosure of Interest:**

None Declared

